# Impact of stigma on people with pre-existing mental disorders during COVID 19 pandemic in Georgia

**DOI:** 10.1192/j.eurpsy.2023.866

**Published:** 2023-07-19

**Authors:** G. Matiashvili

**Affiliations:** Rustavi Mental Health Center, Tbilisi State Medical University, Rustavi; 2 Tbilisi State Medical University, Tbilisi, Georgia

## Abstract

**Introduction:**

From the beginning of the pandemic in Georgia, Covid clinics and Covid hotels were not ready to receive and manage people confirmed Covid 19 with pre-existing mental disorders, because of stigma. As a result of this, the Ministry of Health created special Covid-Psychiatric clinics.

**Objectives:**

Study the impact of stigma on people with pre-existing mental disorders during Covid 19 pandemic. Also clinical, and dynamic characteristics of Covid 19 infection in people with severe mental illness.

**Methods:**

A retrospective statistical analysis of medical records of all hospitalized patients (total 301) at the specialized Rustavi Covid –Psychiatric clinic from November 23, 2020, to March 15, 2022, according to the following parameters: age, gender, mental disorder diagnosis, comorbid chronic illness, vaccination rates, degree of covid infections ongoing, and outcome.

**Results:**

57% of patients were men. Average age-44 years. 67% were asymptomatic or mild with covid symptoms, 25% with moderate severity and 8% were referred to the intensive care unit. 56% of referred patients were hospitalized already in serious conditions. 46% of patients had schizophrenia spectrum disorders. Among referred patients, 60% were women, and 25% had comorbid diabetes mellitus. 44% of patients transferred to the intensive care unit died. Most of the hospitalized patients with pre-existing mental disorders were in remission, and only 7% were referred to other psychiatric clinics for continuing their inpatient treatment.

**Conclusions:**

The study showed that only due to the lack of readiness of the relevant structures, individuals with Covid 19 infection were hospitalized in psychiatric clinics because of their past mental history. Vaccination rates were extremely low than in the general population. The aforementioned indicates a high degree of stigma in the country that makes obstacles for people with mental disorders from receiving adequate and timely medical care. Comorbidities played a big role in degree of COVID infections ongoing, especially diabetes. Statistics also showed that the majority of hospitalized patients did not require inpatient treatment and covid infection did not aggravate the course of most severe mental disorders.

**Disclosure of Interest:**

None Declared

